# Examining the Evolution of the Regulatory Circuit Controlling Secondary Metabolism and Development in the Fungal Genus *Aspergillus*

**DOI:** 10.1371/journal.pgen.1005096

**Published:** 2015-03-18

**Authors:** Abigail L. Lind, Jennifer H. Wisecaver, Timothy D. Smith, Xuehuan Feng, Ana M. Calvo, Antonis Rokas

**Affiliations:** 1 Department of Biomedical Informatics, Vanderbilt University Medical Center, Nashville, Tennessee, United States of America; 2 Department of Biological Sciences, Vanderbilt University, Nashville, Tennessee, United States of America; 3 Department of Biological Sciences, Northern Illinois University, DeKalb, Illinois, United States of America; University College Dublin, IRELAND

## Abstract

Filamentous fungi produce diverse secondary metabolites (SMs) essential to their ecology and adaptation. Although each SM is typically produced by only a handful of species, global SM production is governed by widely conserved transcriptional regulators in conjunction with other cellular processes, such as development. We examined the interplay between the taxonomic narrowness of SM distribution and the broad conservation of global regulation of SM and development in *Aspergillus*, a diverse fungal genus whose members produce well-known SMs such as penicillin and gliotoxin. Evolutionary analysis of the 2,124 genes comprising the 262 SM pathways in four *Aspergillus* species showed that most SM pathways were species-specific, that the number of SM gene orthologs was significantly lower than that of orthologs in primary metabolism, and that the few conserved SM orthologs typically belonged to non-homologous SM pathways. RNA sequencing of two master transcriptional regulators of SM and development, *veA* and *mtfA*, showed that the effects of deletion of each gene, especially *veA*, on SM pathway regulation were similar in *A*. *fumigatus* and *A*. *nidulans*, even though the underlying genes and pathways regulated in each species differed. In contrast, examination of the role of these two regulators in development, where 94% of the underlying genes are conserved in both species showed that whereas the role of *veA* is conserved, *mtfA* regulates development in the homothallic *A*. *nidulans* but not in the heterothallic *A*. *fumigatus*. Thus, the regulation of these highly conserved developmental genes is divergent, whereas–despite minimal conservation of target genes and pathways–the global regulation of SM production is largely conserved. We suggest that the evolution of the transcriptional regulation of secondary metabolism in *Aspergillus* represents a novel type of regulatory circuit rewiring and hypothesize that it has been largely driven by the dramatic turnover of the target genes involved in the process.

## Author Summary

Filamentous fungi produce a highly diverse cadre of secondary metabolites, small molecules whose potent toxic activities are integral to the fungal lifestyle. Most secondary metabolites are narrowly taxonomically distributed, whereas the transcriptional regulators that control their production, alongside with controlling other key processes such as development, are broadly conserved. To gain insight into the evolution of the regulatory circuit governing secondary metabolism and development, we examined the evolution of the genes and pathways underlying these processes as well as the evolution of their transcriptional regulation in the filamentous fungal genus *Aspergillus*, a prolific and important producer of secondary metabolites. We discovered that although secondary metabolic genes and pathways are poorly conserved across *Aspergillus*, their regulation is largely conserved. In contrast, the regulation of highly conserved developmental genes is divergent. These results point to a new type of rewiring that has occurred during the evolution of the regulatory circuit governing secondary metabolism and development in *Aspergillus* in which conserved regulators control a conserved biological process (secondary metabolism), even though the underlying genes and pathways that make up the biological process are not themselves conserved.

## Introduction

Filamentous fungi produce diverse repertoires of small molecules known as secondary metabolites (SMs) [1]. SMs include widely used pharmaceuticals such the antibiotic penicillin [2], the cholesterol-reducing drug lovastatin [3], and the immunosuppressant cyclosporin [4], as well as potent mycotoxins, such as aflatoxin [5] and fumonisin [6,7]. SMs play key ecological roles in territory establishment and defense, communication, and virulence [8–12].

The genes involved in fungal SM pathways are often physically linked in the genome, forming contiguous SM gene clusters [13]. These gene clusters are typically characterized by a backbone gene, such as those encoding nonribosomal peptide synthetases (NRPSs), polyketide synthases (PKSs), hybrid NRPS-PKS enzymes, and prenyltransferases, whose protein products are responsible for synthesizing the proto-SM. Additional genetic components of SM gene clusters include genes for one or more tailoring enzymes that chemically modify SM precursors, transporter genes responsible for exporting the final product, and transcription factors that drive expression of the remaining genes in the gene cluster. For example, the gene cluster responsible for the synthesis of the mycotoxin gliotoxin in the opportunistic human pathogen *Aspergillus fumigatus* contains 13 genes including a non-ribosomal peptide synthase (*gliP*), multiple tailoring enzymes (*gliI*, *gliJ*, *gliC*, *gliM*, *gliG*, *gliN*, *gliF*), a transporter gene (*gliA*), a transcription factor (*gliZ*), and a gliotoxin oxidase gene that protects the fungus from the harmful effects of gliotoxin (*gliT*) [14,15].

Filamentous fungi exhibit a huge amount of SM biochemical diversity. Individual SMs are often known to be produced by only one or a handful of species, and the SM chemotypic profiles of closely related fungi are typically non-overlapping [1,16]. For example, the meroterpenoid fumagillin, originally isolated from *A*. *fumigatus*, has only been detected in *A*. *fumigatus* and some isolates of *Penicillium raistrickii* [17,18]. The gene cluster required for its production appears to be conserved in the *A*. *fumigatus* close relative, *Aspergillus fischerianus*, though only intermediate compounds have been detected from cultures of this and other closely related species [19–21]. In some genera, including *Aspergillus* [22], the extent of fungal SM distribution is so taxonomically narrow that SM chemotypic profiles have been used as unequivocal species-level identifiers.

As might be expected given their key roles in fungal ecology, SM production–and as a consequence SM gene cluster transcriptional activity–is tightly controlled by a complex network of master SM regulators triggered by a wide variety of environmental cues such as temperature, light, pH, and nutrient availability [23]. Among the master SM regulators identified to date are members of the fungal-specific Velvet protein family, which regulate SM production in a light-dependent manner in the model filamentous fungus *Aspergillus nidulans* [24–27]. The founding member of the Velvet family, VeA, stimulates production of diverse types of SMs in various fungal genomes under dark conditions, and has been shown to regulate gliotoxin, fumagillin, fumitremorgin G, and fumigaclavine C gene cluster expression and metabolite production in *A*. *fumigatus* [28]. Recently, a VeA-dependent regulator of secondary metabolism, MtfA, was identified in *A*. *nidulans*, which–unlike VeA–is localized in the nucleus regardless of light conditions [29]. MtfA regulates terrequinone, sterigmatocystin, and penicillin in *A*. *nidulans*; in *A*. *fumigatus*, MtfA is necessary for normal protease activity, and virulence assays using the moth *Galleria mellonella* suggest it plays a role in pathogenicity [30].

In addition to regulating SM, both of these regulators have been linked to the regulation of asexual and sexual development. Timing of SM production with developmental changes is well established in filamentous fungi, and the presence/absence of certain SMs has been linked with developmental changes [31–33]. It has been suggested that regulators that coordinate SM and development allow filamentous fungi to support more “complex” lifestyles through the production of a much greater diversity of natural products than their unicellular yeast relatives, which lack *veA* as well as backbone synthesis genes necessary for SM production [33–35].

Remarkably, both *veA* and *mtfA* appear to be broadly conserved in filamentous fungi with non-overlapping SM profiles [29,36]. We used four well-studied organisms from the fungal genus *Aspergillus*, a highly diverse genus and producer of some of the most iconic SMs, including gliotoxin and penicillin, to investigate the evolutionary variability in the distribution of SM gene clusters and its interaction with these two broadly conserved global transcriptional regulators that differ in their response to light, *veA* and *mtfA*. Our evolutionary analyses show that although both the SM gene clusters as well as their gene content are poorly conserved between *Aspergillus* species, explaining the narrow taxonomic distribution and distinctiveness of their SM profiles, the effects of the global transcriptional regulators on SM production in response to environmental cues are largely conserved across these same species. In contrast, examination of the role of *veA* and *mtfA* in development, a process that involves genes that are highly conserved between the two species and whose regulation is intimately linked to SM regulation, yields a different pattern; whereas the role of *veA* is conserved, *mtfA* regulates development in the homothallic *A*. *nidulans* but not in the heterothallic *A*. *fumigatus*.

Although rewiring has been well documented in yeast and animal regulatory networks [37–44] these studies largely concern rewirings of regulatory signals between otherwise conserved regulators and target genes. Our finding that VeA, and to a certain extent MtfA, regulate non-homologous SM pathways in both *A*. *fumigatus* and *A*. *nidulans* suggests a new type of rewiring in which conserved regulators control a conserved biological process, even though the underlying genes and pathways that make up the biological process are not themselves conserved.

## Results

### The majority of SM gene clusters in *Aspergillus* are species-specific

**Fig 1:**
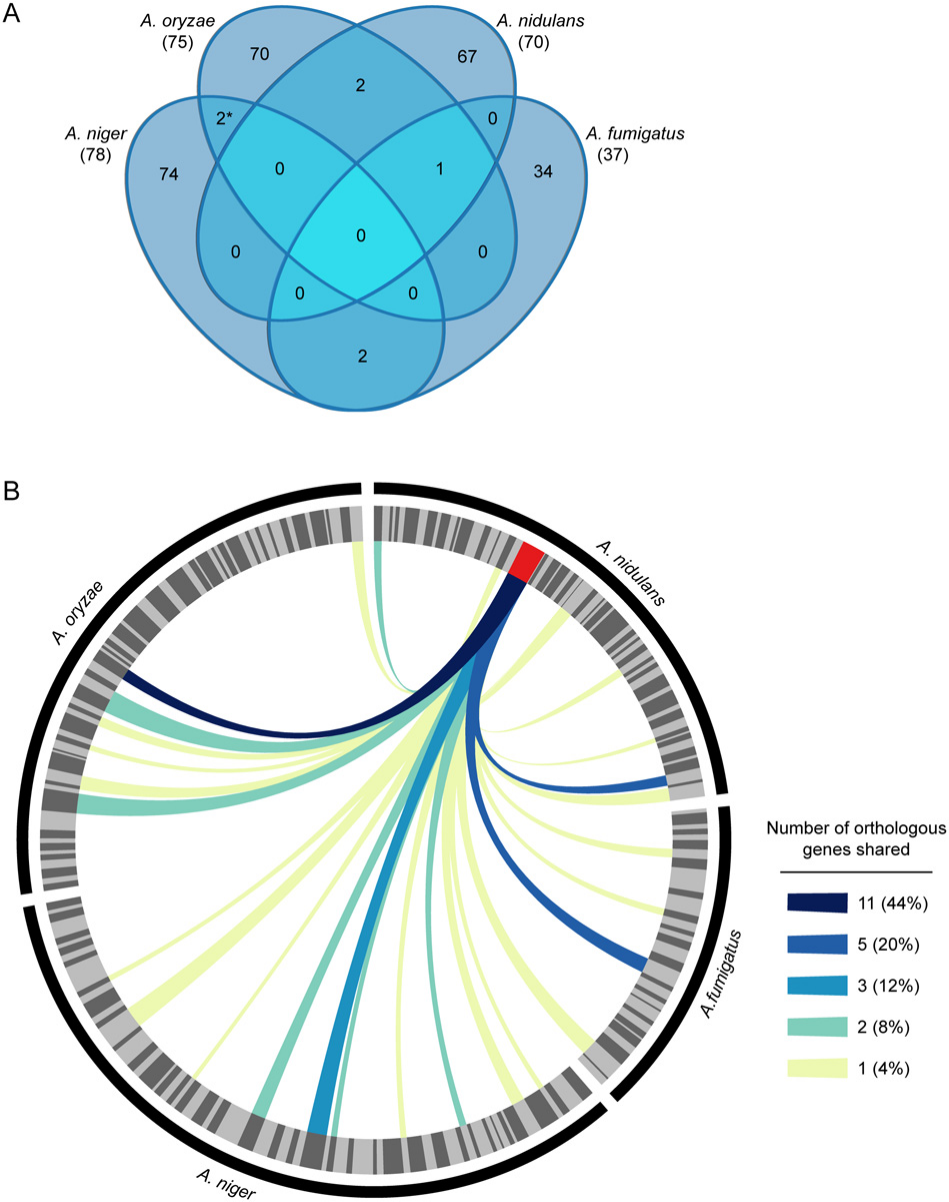
SM gene clusters in *Aspergillus* are not evolutionarily conserved. **A.** Venn diagram showing homologous SM gene clusters between *A*. *fumigatus*, *A*. *nidulans*, *A*. *niger*, and *A*. *oryzae*. Two SM gene clusters were considered homologous if greater than 50% of their genes were orthologs. Numbers in parenthesis indicate the total number of SM gene clusters present in each species. The asterisk (*) denotes that two SM gene clusters in *A*. *oryzae* are homologous to one gene cluster in *A*. *niger* (S2 Table). **B.** Circos plot showing the evolutionary conservation of the *A*. *nidulans* sterigmatocystin gene cluster, one of the most highly conserved SM gene clusters in our study, across *Aspergillus*. in *A*. *fumigatus*, *A*. *nidulans*, *A*. *niger*, and *A*. *oryzae*. The outer black track shows the relative SM gene counts in *A*. *fumigatus*, *A*. *nidulans*, *A*. *niger*, and *A*. *oryzae*. SM gene clusters in each species are indicated by the alternating light and dark grey wedges of the inner track; wedge thickness is proportional to number of clustered genes. The sterigmatocystin gene cluster in *A*. *nidulans* is colored red. Links indicate SM clusters containing one or more genes assigned to the same orthogroup as gene(s) in the sterigmatocystin gene cluster (S3 Table); link color indicates the number of shared genes.

The genomes of *A*. *fumigatus*, *A*. *nidulans*, *Aspergillus oryzae*, and *Aspergillus niger* contain 317, 498, 725, and 584 secondary metabolic genes, respectively, which are organized in 37, 70, 75, and 78 corresponding secondary metabolic gene clusters (S1 Table) [45]. We considered SM gene clusters to be conserved between species if greater than half of the genes in the larger gene cluster were orthologous to greater than half of the genes in the smaller gene cluster. Even with this very liberal definition of gene cluster conservation, we found that no SM gene clusters were conserved across all four species. Moreover, 91.9–96.1% of SM gene clusters were specific to each species, with only 7 SM gene clusters conserved between any species (Fig. 1A, S2 Table). Only one two-gene cluster is completely conserved between two species (*A*. *fumigatus* 27 and *A*. oryzae 9). Although none of these conserved SM gene clusters have chemically characterized products, the fact that only one of the 7 gene clusters appear to be 100% conserved in only two of the four *Aspergillus* species, suggests that the SM chemotypic profiles of the four species are non-overlapping.

While only one (at the 100% level) or very few (at the 50% level) conserved SM gene clusters can be identified in comparisons between any of these four species, SM gene clusters do contain genes whose orthologs are parts of other, non-homologous, SM gene clusters. For example, the 25 genes in the sterigmatocystin gene cluster in *A*. *nidulans*, one of the largest SM gene clusters present in the genomes analyzed, have orthologs in 25 SM gene clusters in the other three species as well as inparalogs in 8 other *A*. *nidulans* SM gene clusters (Fig. 1B, S3 Table). However, in all but one case, less than 20.0% (5 genes) of the sterigmatocystin gene cluster is present in the other gene cluster. The only exception is the truncated aflatoxin gene cluster of *A*. *oryzae*, which shares 11 orthologs with the ST gene cluster. Although the *A*. *oryzae* aflatoxin gene cluster is non-functional [46–48], the evolutionary conservation between the aflatoxin and sterigmatocystin gene clusters is reflected in the fact that sterigmatocystin is the penultimate precursor product of the aflatoxin biosynthetic pathway [49].

### *Aspergillus* SM genes are significantly less conserved than genes for primary metabolism

Given the remarkable lack of conservation of SM gene clusters between *Aspergillus* species, we next tested whether the genes belonging to these clusters were also less conserved by comparing their degree of evolutionary conservation to that of genes involved in primary metabolism. To determine the percentage of species-specific orthogroups (see Methods) involved in SM and primary metabolism, we first determined the number of orthogroups with genes annotated to these functional categories that contained no genes in any other species (i.e., all orthogroups that contain only a single gene or only putative inparalogs). To determine the conservation of gene function across species, we determined the number of orthogroups in each genome that are either entirely species-specific or whose members in other genomes are annotated to different functional categories.

**Fig 2:**
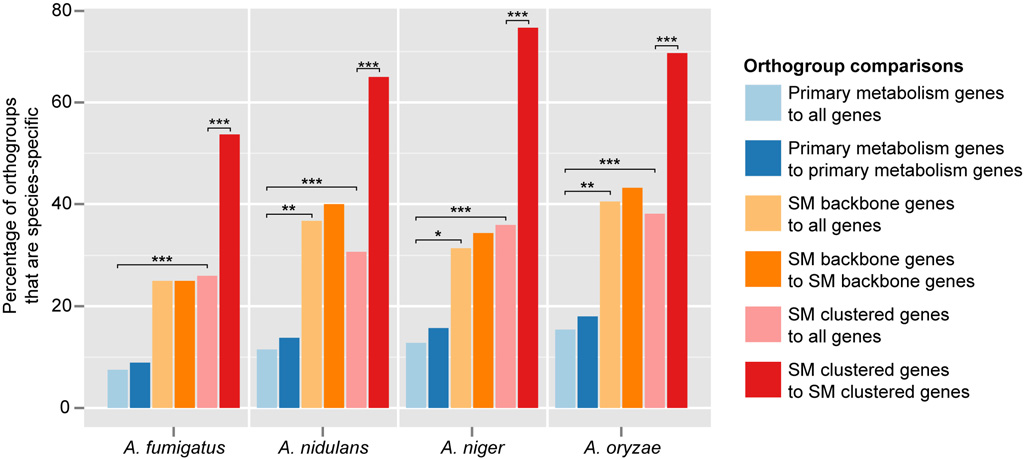
Many more *Aspergillus* SM genes are species-specific than primary metabolic genes. For each species, dark blue bars indicate the percentage of primary metabolism orthogroups that is species-specific when compared to all genes annotated as participating in primary metabolism in the other three species; light blue bars indicate the percentage of each species’ primary metabolism orthogroups that is species-specific compared to all genes, irrespective of their annotation, in the other three species. Similarly, light orange bars indicate the percentage of each species’ SM backbone synthesis orthogroups that is species-specific when compared to all genes annotated as SM backbone synthesis genes in the other three species; dark orange bars indicate the percentage of each species’ SM backbone synthesis orthogroups that is species-specific compared to all other genes. Light red bars indicate the percentage of each species’ clustered SM orthogroups that is species-specific when compared to all genes annotated as clustered SM genes in the other three species; dark red bars indicate the percentage of each species’ clustered SM orthogroups that is species-specific compared to all other genes. Asterisks indicate statistically significant differences based on a *P-*value ≤ 0.01 (*), ≤ 0.001 (**), or ≤ 0.0001 (***) in a two-tailed Fisher’s exact test (S4 Table).

We found that SM orthogroups were significantly far less conserved than primary metabolic orthogroups in all four genomes examined (adjusted *P <* 1e^-10^ for all combinations; S4 Table). Specifically, the percentage of species-specific primary metabolic orthogroups ranged between 7.5 and 15.4% (Fig. 2). When we considered conservation of function (i.e. whether orthogroups contained genes annotated to primary metabolism), we saw a slight and non-significant increase in the percentage of species-specific orthogroups (8.9–18%; Fig. 2). In contrast, 25.0% to 40.5% of orthogroups containing SM backbone genes were species-specific; considering functional conservation had a negligible impact on these percentages (Fig. 2). Orthogroups containing genes in SM clusters exhibited similar percentage ranges for species-specificity (26.0–38.1%; Fig. 2). Strikingly, the orthologs of many SM genes in a given species were not in SM gene clusters in any of the other species; specifically, 53.7% (in *A*. *fumigatus*, with 37 SM clusters) to 74.7% (in *A*. *niger*, with 77 SM gene clusters) of orthogroups containing an SM gene from a particular genome had no orthologs in SM clusters in any of the other genomes.

### VeA regulates the same biological processes as well as the same fraction of the genome in both *A*. *nidulans* and *A*. *fumigatus*

We next examined the function of the conserved secondary metabolic regulator VeA by performing RNA sequencing [19,48,50] of Δ*veA* and wild-type (WT) *A*. *fumigatus* strains TSD1.15 [51] and CEA10 and *A*. *nidulans* strains TXFp2.1 and TRV50.2 [52] to identify genes and biological processes that are differentially regulated in Δ*veA* vs WT in the two species. Of the 9,783 transcribed genes in the *A*. *fumigatus* genome, 1,546 (15.8%) were over-expressed and 1,555 (15.9%) were under-expressed in the Δ*veA* vs WT analysis in *A*. *fumigatus* (Tables 1, S5). We observed very similar numbers of genes differentially regulated in the *A*. *nidulans* Δ*veA* vs WT analysis; out of 10,709 genes in the *A*. *nidulans* genome, 1,165 (10.9%) were over-expressed and 1,671 genes (15.6%) were under-expressed. In total, approximately 32% and 26% of protein coding genes were differentially regulated in Δ*veA* compared to WT in *A*. *fumigatus* and *A*. *nidulans*, respectively.

**Table 1:** Differentially expressed genes in Δ*veA* vs WT and Δ*mtfA* vs WT comparisons for *A*. *fumigatus* and *A*. *nidulans*.

Condition	Species	Total dif. expressed^a^	Per. diff. expressed^a^	Under- expressed^a^	Over- expressed^a^
Δ*veA*	*A*. *fumigatus*	3,101	31.7%	1,555	1,546
Δ*veA*	*A*. *nidulans*	2,836	26.5%	1,671	1,165
Δ*mtfA*	*A*. *fumigatus*	97	0.9%	63	34
Δ*mtfA*	*A*. *nidulans*	968	9.0%	568	400

^a^Number of differentially expressed genes relative to wild type

**Fig 3:**
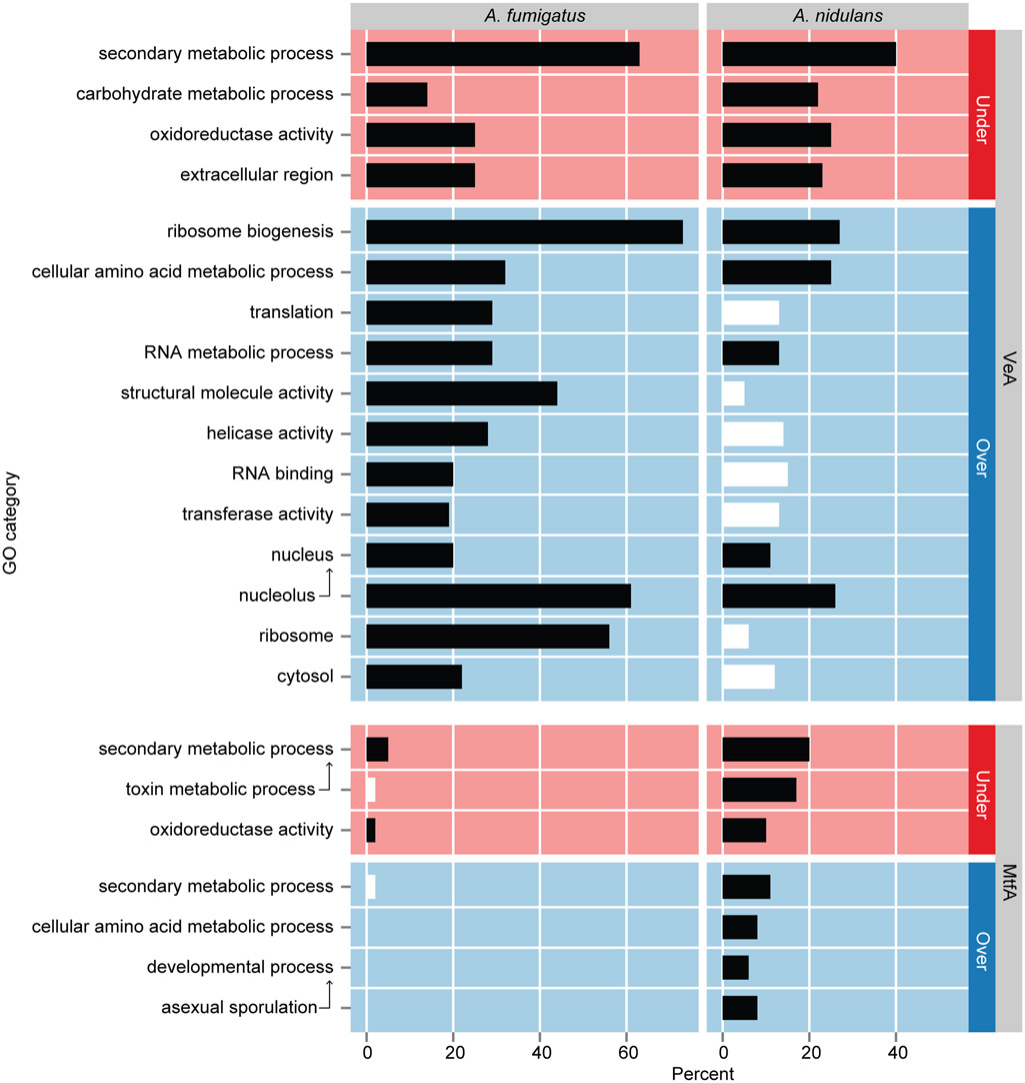
GO term enrichment analysis of genes differentially expressed in Δ*veA* and Δ*mtfA* relative to wild type in *A*. *fumigatus* and *A*. *nidulans*. Statistically overrepresented gene ontology (GO) categories in under-expressed (red) and over-expressed (blue) gene sets in Δ*veA* and Δ*mtfA* relative to wild type. Arrows point to GO term ancestors. Horizontal bars show the percentage of each gene set assigned to a particular GO term with black bars indicating significant enrichment (Benjamini & Hochberg adjusted *P-*value ≤ 0.05 in a hypergeometric test; S6 Table); white bars indicate no significant enrichment.

To characterize the broad biological processes that these differentially regulated genes are involved with, we performed GO term enrichment analysis using the *Aspergillus* GOSlim term hierarchy [53,54]. Remarkably, the same four GO terms, namely secondary metabolic process, carbohydrate metabolic process, oxidoreductase activity, and extracellular region, were significantly enriched in under-expressed genes in both *A*. *nidulans* and *A*. *fumigatus*, showing that VeA is a positive regulator of the same processes in both species (Fig. 3). Over-expressed genes in *A*. *fumigatus* were significantly enriched for twelve GO terms potentially related to cell growth, namely ribosome biogenesis, cellular amino acid metabolic process, translation, rna metabolic process, structural molecule activity, helicase activity, rna binding, transferase activity, nucleus, nucleolus, ribosome, and cytosol. Five of these twelve terms were also significantly enriched in *A*. *nidulans* (ribosome biogenesis, cellular amino acid metabolic process, rna metabolic process, nucleus, and nucleolus). Over-expressed genes were present in the remaining seven terms in *A*. *nidulans* but did not show statistically significant enrichment (S6 Table).

In addition to the enrichment of the GO term secondary metabolic process in the differentially expressed genes from the Δ*veA* vs WT comparison in both *A*. *nidulans* and *A*. *fumigatus*, a large portion of the 317 *A*. *fumigatus* SM cluster genes and 498 *A*. *nidulans* SM cluster genes was also differentially expressed in the Δ*veA* strains. In *A*. *fumigatus*, 98 genes (30.9%) were under-expressed and 38 (12.0%) were over-expressed; in *A*. *nidulans*, 184 genes (36.9%) were under-expressed and 67 (13.5%) were over-expressed (S5 Table). Interestingly, all constituent genes of several SM gene clusters were differentially expressed. For example, all genes in the *A*. *fumigatus* pseurotin A gene cluster and all genes in the *A*. *nidulans* asperthicin cluster were under-expressed (S10 Table).

### MtfA’s regulatory role in secondary metabolism is smaller in scope in *A*. *fumigatus* compared to *A*. *nidulans*

We next examined the role of the recently identified SM regulator MtfA [29,30] in *A*. *fumigatus* and *A*. *nidulans* by performing RNA sequencing and differential gene expression analysis of Δ*mtfA* vs WT strains of both species (*A*. *fumigatus* tTDS4.1 Δ*mtfA* [30] and CEA10, *A*. *nidulans* TRVp Δ*mtfA* and TRV50.2 [29]). In contrast to our findings with *veA*, we found an approximately 10-fold difference in the percentage of genes regulated in both species (Tables 1, S5). Thirty-six genes were over-expressed (0.4%) and 63 (0.6%) were under-expressed in the *A*. *fumigatus* Δ*mtfA* vs WT analysis, whereas in the *A*. *nidulans* Δ*mtfA* vs WT analysis 400 genes were over-expressed (3.7%) and 568 were under-expressed (5.3%).

To determine the functional categories impacted by *mtfA* deletion both species, we performed GO term enrichment analysis on the genes differentially expressed between Δ*mtfA* and WT strains. Under-expressed as well as over-expressed genes in *A*. *nidulans* were significantly enriched for secondary metabolic process, toxin metabolic process and oxidoreductase activity, suggesting that MtfA is involved in positive and negative regulation of different secondary metabolites (Fig. 3). Over-expressed genes in *A*. *nidulans* were also significantly enriched for asexual developmental processes, namely developmental process and asexual sporulation.

Under-expressed genes in *A*. *fumigatus* were significantly enriched for two of the three processes as in *A*. *nidulans*, namely secondary metabolic process and oxidoreductase activity. However, over-expressed genes in *A*. *fumigatus* were not significantly enriched for any GO terms; some over-expressed genes were present in the secondary metabolic process term, though this was not statistically significant (S6 Table).

Examination of the 317 *A*. *fumigatus* SM cluster genes and 498 *A*. *nidulans* SM cluster genes showed that they too were also differentially expressed in both *A*. *fumigatus* and *A*. *nidulans* Δ*mtfA* mutants compared to the WT strains. In *A*. *nidulans*, 107 of the 498 SM cluster genes (21.5%) were under-expressed and 32 (6.4%) were over-expressed (S5 Table). In some SM gene clusters, such as in the *dba* cluster, all genes were under-expressed in Δ*mtfA A*. *nidulans* relative to WT (S10 Table). In contrast, many fewer SM cluster genes were differentially expressed in *A*. *fumigatus* Δ*mtfA*; 32 out of the 317 genes (10.1%) were under-expressed and 4 (1.3%) were over-expressed (S5 Table). Despite this much smaller number of differentially expressed SM genes, at least one entire SM gene cluster (the pseurotin A cluster) was under-expressed in *A*. *fumigatus* Δ*mtfA* relative to ST (S10 Table).

### Transcriptional regulation of similar processes regardless of gene conservation

**Fig 4:**
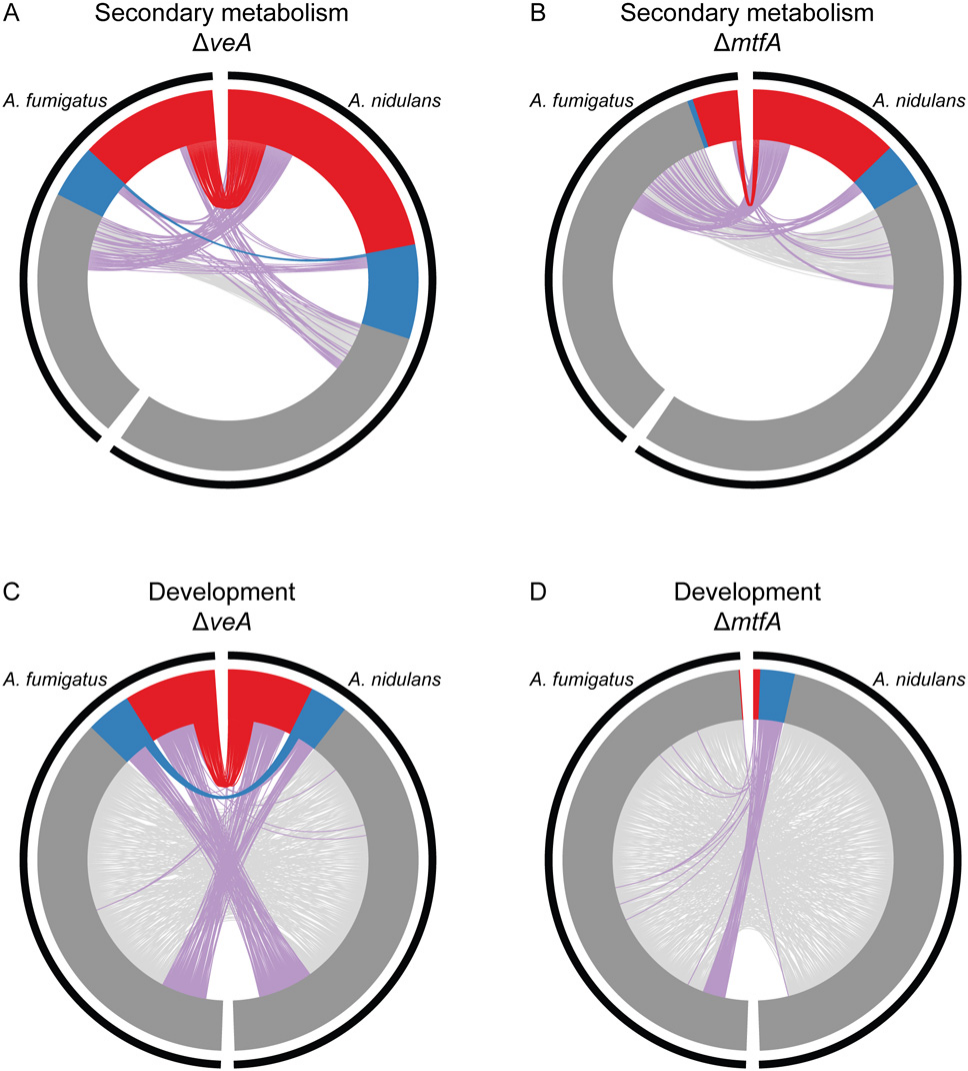
Orthology of SM and development genes differentially expressed in Δ*veA* and Δ*mtfA* relative to wild type in *A*. *fumigatus* and *A*. *nidulans*. Circos plots of genes in SM clusters **(A,B)** and developmental genes **(C,D)** showing change in gene expression patterns in Δ*veA* vs WT **(A,C)** and in Δ*mtfA* vs WT **(B,D)** experiments. In all plots, *A*. *fumigatus* genes are shown on the left and *A*. *nidulans* genes on the right. Inner tracks show the relative number of under-expressed genes (red), over-expressed genes (blue) and not differentially expressed genes (grey) in each species. Links between the two species indicate orthologous genes that are both under-expressed (red links), both over-expressed (blue links) and both not differentially expressed (light grey links); purple links indicate that the orthologous genes have conflicting expression patterns. In both **A** and **B**, the 317 SM cluster genes present in *A*. *fumigatus* are shown on the left and the 498 *A*. *nidulans* SM cluster genes are shown on the right. Many SM cluster genes are differentially expressed in the Δ*veA* vs. WT comparison in both species (**A**). Many SM cluster genes are also differentially expressed in the Δ*mtfA* vs. WT comparison in *A*. *nidulans*; the proportion of differentially expressed genes in the Δ*mtfA* vs. WT comparison in *A*. *fumigatus* is much smaller (**B**). The absence of links between most of the SM cluster genes in the two species indicates that most SM cluster genes are species-specific. For those genes that share orthology in the two species, they often differ in their expression in response to a master transcriptional regulator. In **C** and **D**, the 478 genes annotated to the GO term developmental process in *A*. *fumigatus* are shown on the left side of the plot and the 490 genes annotated to the same GO term in *A*. *nidulans* are shown on the right side. The presence of links between most genes in these two species indicates a high degree of gene conservation. The expression patterns of all individual genes, including those present in SM gene clusters and those involved in developmental processes, are shown in S5 Table. Expression patterns of the SM cluster genes organized by individual SM pathways are shown in S10 Table.

To examine whether gene conservation and involvement in a biological process (secondary metabolism and development) correlated with conservation of regulation by VeA and MtfA, we tested whether orthologous and species-specific genes in *A*. *nidulans* and *A*. *fumigatus* showed the same or different responses in Δ*veA* vs WT and Δ*mtfA* vs WT analyses (Fig. 4). Even though both species contain large numbers of species-specific genes in their SM gene clusters, the proportions of differentially expressed SM cluster genes in both Δ*veA A*. *nidulans* and Δ*veA A*. *fumigatus* were remarkably similar (Figs. 1A, 4A; S5 Table).

Examination of SM cluster genes that are differentially expressed suggests that even when genes in SM clusters have orthologs, these orthologs were often differentially regulated by Δ*veA*. Entire SM pathways that are over- or under-expressed in one species may have orthologs with completely different expression patterns in the other species, as in the case of the *dba* SM gene cluster in *A*. *nidulans* (S2 Fig). Specifically, of the 184 under-expressed SM cluster genes in Δ*veA A*. *nidulans*, 64 genes (34.8%) had an ortholog in *A*. *fumigatus* (S7 Table). Of these 64 genes, 45 (70.3%) had at least one differentially expressed ortholog in *A*. *fumigatus*, and 37 (57.8%) had at least one similarly under-expressed ortholog in *A*. *fumigatus* (S7 Table). Fewer SM genes were over-expressed in either Δ*veA A*. *nidulans* or *A*. *fumigatus*; of the 67 over-expressed genes in *A*. *nidulans*, only 14 (20.9%) had orthologs in *A*. *fumigatus*. Of these 14 genes, 5 had at least one differentially expressed ortholog in *A*. *fumigatus*, and 3 had at least one similarly over-expressed ortholog in *A*. *fumigatus* (S7 Table).

When *mtfA* was deleted, fewer SM cluster genes were differentially expressed in *A*. *fumigatus* than in *A*. *nidulans*. Of the 107 under-expressed genes in Δ*mtfA A*. *nidulans*, 36 (33.6%) had an ortholog in *A*. *fumigatus* (Fig. 4B, S7 Table). Unlike *veA*, however, only 6 of these conserved genes had differentially expressed orthologs in *A*. *fumigatus*. Finally, of the 32 over-expressed genes in Δ*mtfA A*. *nidulans*, 2 of the 12 genes with orthologs in *A*. *fumigatus* had orthologs that were differentially expressed.

Apart from their involvement in the global regulation of SM, both VeA and MtfA are also involved in the regulation of asexual and sexual development. In contrast to genes involved in SM, genes involved in asexual and sexual development in *Aspergillus* have been shown to be highly conserved across the genus [55]. Of the 490 genes annotated to the GO term developmental process in *A*. *nidulans*, 462 have at least one ortholog among the 478 genes annotated to this term in *A*. *fumigatus*. In Δ*veA* A. *nidulans*, 72 developmental genes are under-expressed and 32 are over-expressed. Of the 72 under-expressed genes, 66 (91.7%) have an ortholog in *A*. *fumigatus*; of these 66 orthologs, 30 were differentially expressed in both species (Fig. 4C, S7 Table). There were fewer over-expressed developmental genes in Δ*veA A*. *nidulans*, but they showed similar trends; 31 of the 32 over-expressed genes have an ortholog, 15 of which had differentially expressed orthologs in *A*. *fumigatus* and 11 of which had over-expressed orthologs. In contrast with *veA*, many more developmental genes were differentially expressed in *A*. *nidulans* Δ*mtfA* (35) than in *A*. *fumigatus* Δ*mtfA* (1). While 4 of the 6 under-expressed genes and 28 of the 29 over-expressed genes in *A*. *nidulans* had orthologs in *A*. *fumigatus*, none of these orthologs were differentially expressed (Fig. 4D, S7 Table).

## Discussion

To gain insight into the evolution of the regulatory circuit governing SM and development in filamentous fungi, we examined the evolution of their underlying genes and pathways as well as the evolution of their regulation by two global transcriptional regulators in *Aspergillus*, a genus whose members prolifically produce diverse secondary metabolites. We discovered that although secondary metabolic genes and pathways are poorly conserved, their regulation by VeA, and to a certain extent by MtfA, is largely conserved. In contrast, the MtfA regulation of highly conserved developmental genes is divergent, whereas that of VeA is conserved. Below, we discuss the significance of our results with respect to the evolution of SM gene clusters as well as to the evolution of the regulatory circuit controlling secondary metabolism and development in filamentous fungi.

### *Aspergillus* secondary metabolic genes and gene clusters are largely species-specific

All but one of the SM gene clusters present in the four *Aspergillus* species we examined were species-specific. Even when we used a very low threshold of 50% evolutionary conservation, we found that there were no clusters conserved in all four species, one cluster conserved in three species, and a very small number conserved between pairs of species (Fig. 1A). Consistent with our results, an examination of the close relatives *A*. *fumigatus*, *A*. *fischerianus*, and *Aspergillus clavatus*, using an 80% threshold of evolutionary conservation, also found relatively small numbers of conserved SM gene clusters [56]. Remarkably, variation in SM gene cluster content can sometimes be even strain-specific; for example, a single SM gene cluster has been shown to vary in its presence in *A*. *fumigatus* isolates [57], and several SM gene clusters also appear to vary between isolates of *A*. *niger* [58].

Surveys of fungal genomes show that for any given fungus there are many more gene clusters than known SMs, suggesting that the currently characterized SMs might be only a small fraction of the SMs that a fungus can produce [56,59]. For example, 33 of the 37 putative SM gene clusters in *A*. *fumigatus* have no characterized products, despite evidence from metabolomics surveys suggesting that the fungus produces many SMs [19,45,60]. Interestingly, most known SMs are produced by only a handful of species [16] and SM profiles between closely related species are quite distinct [17,61], both indications that the typical taxonomic distribution of SMs is narrow. Thus, the observed extremely high degree of taxonomic narrowness of SM gene clusters is not only consistent with the distribution of known SMs, but also suggests that this distribution is likely to be typical of all the SMs produced by a fungus.

SM gene clusters are largely species-specific, so one hypothesis is that their constituent genes are also species-specific. Another alternative is that their constituent genes are conserved but have been reshuffled and become members of different SM gene clusters in different species. Our analysis shows support for both hypotheses. For example, we found that a much larger fraction of the genes comprising SM gene clusters is species-specific than of genes participating in primary metabolism (Fig. 2), which is likely explained by extensive gene duplication and loss [35,62,63] *de novo* gene emergence [64], horizontal gene transfer [65–69], as well as by very high sequence divergence. However, a considerable fraction of SM genes was not species-specific. In several cases, the orthologs of SM genes in one *Aspergillus* species were found residing in non-homologous SM pathways in another species; for example, the 25 genes in the sterigmatocystin gene cluster in *A*. *nidulans* have orthologs in 25 distinct SM gene clusters in the other three species (Fig. 1B). Surprisingly, the orthologs of many genes that resided within an SM gene cluster in one species were not in an SM gene cluster in another species (Fig. 2). The majority of these orthologs either lack annotation or are annotated as being involved in primary metabolism, consistent with a model in which the gene content of SM gene clusters is formed or altered through the recruitment and incorporation of native genes involved in primary metabolism and other essential cellular processes.

### The evolution of the circuit regulating secondary metabolism and development in *Aspergillus*

By comparing genome-wide gene expression of deletion mutants of *veA* and *mtfA* with wild-type strains in both *A*. *fumigatus* and *A*. *nidulans* we assessed the degree to which their regulatory roles in controlling secondary metabolism and development are conserved (Fig. 4A and 4C). The involvement of VeA in regulating both processes is highly conserved in both *A*. *fumigatus* and *A*. *nidulans*. Remarkably, however, the downstream SM genes regulated by VeA are different between the two species; this difference is not fully accounted by the fact that VeA regulates many species-specific SM genes. Many genes that are regulated by VeA in one species are present but are not regulated by VeA in the other (Fig. 4A).

This conservation in regulatory logic (i.e., conservation in the regulation of secondary metabolism) despite the dramatic change in the genes involved may be explained by considering the number and complexity of VeA’s interacting partners as well as VeA’s putative transcription factor function, which in combination offer many degrees of freedom for changes in the regulation of specific SM genes and pathways in either species. Specifically, VeA is known to have many interacting partners [33] including the Velvet family protein VelB, which it transports from the cytoplasm to the nucleus, where both proteins form a trimeric complex with LaeA that regulates secondary metabolism production and development [24]. Furthermore, VeA also functions outside of this complex [24,28]; it interacts with red light-sensing proteins in the nucleus [27] and with other methyltransferases [70], and it has been hypothesized that it may also act as a scaffold protein for the recruitment of additional transcriptional regulators [33,36]. Finally, recent analysis has shown that the Velvet domain is a DNA-binding domain, and that Velvet family proteins may act as direct transcriptional regulators [71].

The involvement of MtfA in regulating SM in both *A*. *fumigatus* and *A*. *nidulans* is also conserved, although the numbers of SM genes that are under its control differ considerably between the two species. As with *veA*, comparing expression patterns between Δ*mtfA* and WT identified differentially expressed species-specific and conserved SM genes. Again like *veA*, many conserved genes that are regulated by MtfA in one species are not regulated in the other (Fig. 4B). This rewiring could either be due to a divergence in the signal that targets these genes for regulation by MtfA, a C2H2 zinc finger transcription factor [29], or its interacting partners. Although not much is known about MtfA’s interacting partners, indirect support for their presence comes from our finding that genes involved in SM are significantly over-represented in both the under-expressed and the over-expressed gene sets in *A*. *nidulans* (Fig. 3), suggesting that the regulatory effect of MtfA in certain regulatory partnerships is positive and in others negative. Interestingly, our results also suggest that MtfA genetically interacts with (and acts downstream of) VeA in *A*. *nidulans*, but not in *A*. *fumigatus* [28–30], as its expression is decreased in Δ*veA* vs WT in *A*. *nidulans* but not in Δ*veA* vs WT in *A*. *fumigatus* (S5 Table). Finally, MtfA appears to regulate development in the homothallic *A*. *nidulans*, but not in the heterothallic *A*. *fumigatus* (Figs. 3,4). Unlike *veA*, deleting *mtfA* influenced the expression of developmental genes only in *A*. *nidulans* but not in *A*. *fumigatus*. This is consistent with the loss in *A*. *fumigatus* or gain in *A*. *nidulans* of the signal that directs MtfA or its downstream targets to regulate developmental processes.

VeA’s central role in coordinating SM and development under dark conditions as well as its large number of interacting partners likely explain in part why many more genes are differentially expressed in its absence in both *A*. *nidulans* and *A*. *fumigatus* than in the absence of MtfA. Gaining a more complete understanding of the molecular mechanisms through which VeA and MtfA regulate downstream targets is an interesting line of future inquiry that harbors significant promise in elucidating how VeA and MtfA have been rewired in different fungal species. However, it is likely that VeA and MtfA globally regulate SM and developmental processes not only directly but also indirectly, through interactions with other regulatory proteins; thus, obtaining a full mechanistic understanding of the rewiring of this regulatory circuit will likely also require characterization of VeA and MtfA’s interacting partners and their regulatory functions.

### A novel type of regulatory circuit rewiring?

It is abundantly clear that changes in transcriptional regulation, also known as rewiring, are a major driver of phenotypic divergence [40,43]. It is also becoming increasingly clear that rewiring of regulatory circuits can also take place in the absence of phenotypic change [42,72,73]. Irrespective of whether it leads to phenotypic change or not, rewiring is typically thought to occur either through changes to the transcriptional regulators or through changes to the regulatory signals between the regulators and the target genes.

An example of rewiring due to changes to the transcriptional regulator is offered by the galactose pathway, which is controlled by Gal4p in the baker’s yeast *Saccharomyces cerevisiae*, but by the non-related Cph1p in the human commensal *Candida albicans* [37,74]. The acquisition or loss of specific motifs in otherwise conserved transcriptional regulators can also lead to rewiring [75,76], as can acquisition or loss of interacting regulatory partners [41]. An example of changes in regulatory signals is offered by the small percentage of Mcm1 regulated genes shared between *S*. *cerevisiae* and *C*. *albicans* [77], which can be largely attributed to high rates of *cis*-regulatory sequence gain and loss that Mcm1 binds to. This is likely to be the major explanation in cases that involve conserved regulators, conserved target genes, and conserved phenotypes [78,79].

**Fig 5:**
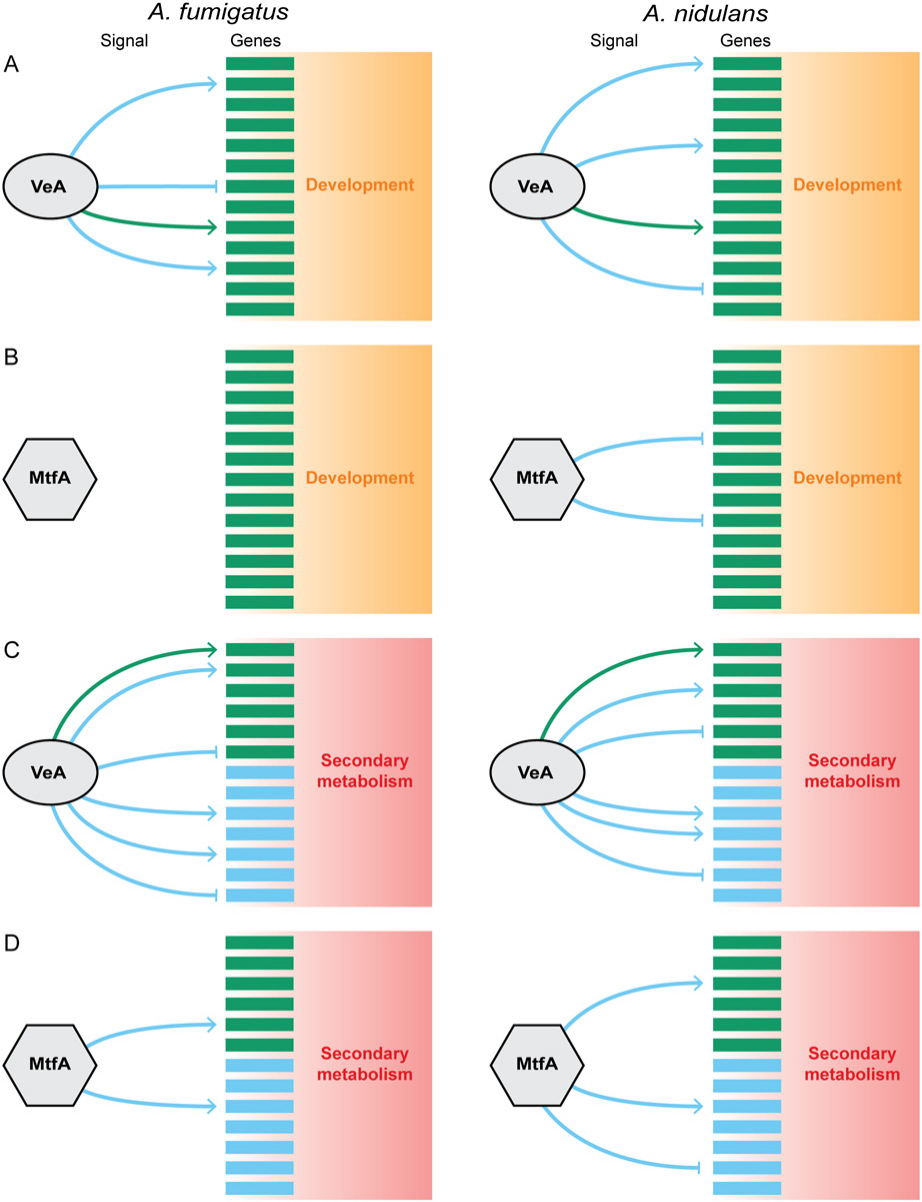
Model of the evolution of the regulatory circuit controlling secondary metabolism and development in *Aspergillus*. Generalized gene regulatory networks for development **(A,B)** and secondary metabolism **(C,D)** for the transcriptional regulators VeA **(A,C)** and MtfA **(B,D)** in *A*. *fumigatus* and *A*. *nidulans*. As master transcriptional regulators, VeA and MtfA can promote the expression of a gene (indicated by an arrow →) or suppress it (indicated by a bar ⟞). Target genes are either conserved (i.e., present in both species; green squares) or not (i.e., species-specific; blue squares). Similarly, the signal emanating from each transcriptional regulator that coordinates target gene regulation is either conserved (i.e., acting in the same fashion on genes conserved in both species; green arrows/bars) or not (i.e., acting in different fashion on genes conserved in both species or acting on species-specific genes; blue arrows/bars). Both transcriptional regulators contribute to the regulation of secondary metabolism in both *A*. *fumigatus* and *A*. *nidulans* even though the underlying genes in the two species are largely non-homologous **(C,D)**. In contrast, the role of these two regulators in development, where most underlying genes are conserved in both species, is different; the role of *veA* is conserved, but *mtfA* regulates development in the homothallic *A*. *nidulans* but not in the heterothallic *A*. *fumigatus* **(A,B)**.

Although the evidence for the regulatory impact of VeA and MtfA on secondary metabolism and development is genetic in our case (i.e., it is not known whether VeA or MtfA directly bind to target gene cis-regulatory elements or whether they control their expression indirectly), the evolution of VeA and MtfA regulation on development is consistent with rewiring that involves changes either to the transcriptional regulator or the regulatory signals between the regulator and the target genes (Fig. 5A,B). In the case of VeA, the rewiring is not associated with phenotypic change–genes involved in development are regulated by VeA in both *A*. *fumigatus* and *A*. *nidulans*, whereas in the case of MtfA the rewiring might be associated with the differences in sexual and asexual development between the two species [55,80].

In contrast to the rewiring examples mentioned so far, the major driver of change in the evolution of VeA and MtfA regulation on SM appears to be the dramatic turnover in the target genes involved in the process, which has resulted in their high degree of species specificity and their even higher species-specific presence in SM gene clusters (Figs. 2 and 5C,D). Although it seems reasonable to exclude the transcriptional regulators as potential co-drivers of the rewiring because the key domains of both VeA and MtfA are conserved across *Aspergillus* [29,36], it is possible that the regulatory signals or the differential presence or absence of interacting regulatory partners may have also contributed to rewiring. However, given the vital ecological importance of secondary metabolites in fungal ecology, it seems reasonable to hypothesize that the evolution of the regulatory circuit governing secondary metabolism in filamentous fungi is primarily driven by the likely extreme evolutionary pressure imposed on each fungus to produce its own unique blend of secondary metabolites.

## Materials and Methods

### Genome sequences and orthogroup definitions for *A*. *nidulans*, *A*. *fumigatus*, *A*. *oryzae*, and *A*. *niger*

All genome sequences and annotations for *A*. *nidulans* FGSC A4 s10-m02-r03, *A*. *fumigatus* AF293 s03-m04-r11, *A*. *oryzae* RIB40 s01-m08-r21 and *A*. *niger* CBS 513.88 s01-m06-r10 were taken from the *Aspergillus* Genomes Database (*Asp*GD) [54]. Groups of orthologous genes (orthogroups) for these four genomes were taken from *Asp*GD’s orthology assignments for 16 *Aspergillus* species, which were generated using a Jaccard clustering approach [81]. *Asp*GD orthogroups contain groups of genes that are thought to have descended from the *Aspergillus* common ancestor; genes from the same species that are part of a given orthogroup are defined as in-paralogs that have duplicated at some later point after the species diverged from the *Aspergillus* common ancestor. Species-specific genes, which were absent from *Asp*GD orthogroups, were organized into species-specific orthogroups using the MCL algorithm in combination with all-versus-all protein BLAST search [82]. Proteins with BLAST hits with 60% query and subject coverage, an e-value of less than 1e^-5^, and a percent identity of greater than 60% were subsequently clustered in MCL with an inflation parameter of 2 and were considered species-specific orthogroups. Proteins that did not pass the BLAST cutoffs were considered single-gene, species-specific orthogroups.

### Gene category definitions

Genes involved in secondary metabolism were taken from a previous study that expertly annotated secondary metabolic gene clusters in the four species under study [45]. Manually curated gene cluster boundaries were used when available. Primary metabolism genes were annotated using a previously described enzyme classification pipeline which utilizes KEGG Enzyme Commission annotations [62]. Genes involved in development were determined from all genes in *A*. *fumigatus* and *A*. *nidulans* annotated to the GO term developmental process (GO:0032502) in AmiGO [83]. This data was accessed on 2014–07–19.

### Strains and culture conditions

The strains used in this study include *A*. *fumigatus* CEA10, TSD1.15(Δ*veA*) and TTDS4.1(Δ*mtfA*) [30,51] and *A*. *nidulans* TRV50.2 [52], TXFp2.1(Δ*veA*) generated in this study, and TRVpΔ*mtfA* [29]. Deletion and wild-type strains presented isogenic genetic backgrounds, and all strains used in this study are prototrophs. Many *A*. *nidulans* studies have used a *veA* partial deletion [84]. For the present study we generated a strain with a complete deletion of the *veA* coding region, TXFp2.1(Δ*veA*). This strain was constructed as follows. First, The *veA* deletion cassette was obtained by fusion PCR as previously described [85]. A 1.4 kb 5’ UTR and a 1 kb 3’ UTR *veA* flanking regions were PCR amplified from wild type FGSC4 genomic DNA with primers veA_comF and AnidveA_p2, and ANVeASTagP3 and ANVeASTagP4 primers sets, respectively (S8 Table). The *A*. *fumigatus pyrG* (*pyrG* ^*A*.*fum*^) selectable marker was amplified with AnidveA_p5 and ANVeASTagP6 primers from plasmid p1439. The 5’ and 3’ UTR fragments were then PCR fused to *pyrG* ^*A*.*fum*^ to generate the *veA* replacement construct using primers AnidveA_P7 and AnidveA_P8. The deletion cassette was transformed into *A*. *nidulans* RJMP1.49 strain [86]. The resulting colonies were then transformed with the pSM3 plasmid containing the *A*. *nidulans pyroA* to generate a prototroph with a Δ*veA* background. This strain was confirmed by DNA analysis and designated as TXFp2.1.

All strains were grown in liquid stationary cultures in Czapek-Dox medium (Difco) in the dark. The experiments were carried out with two replicates. After 72 hours of incubation at 37°C mycelial samples were harvested, immediately frozen in liquid nitrogen and lyophilized.

### RNA extraction

Total RNA was isolated from lyophilized mycelia using the directzol RNA MiniPrep Kit (Zymo) according to the manufacturer’s instructions. RNA then was quantified using a nanodrop instrument. Expression patterns of *veA* and *mtfA* were verified in the *A*. *fumigatus* and *A*. *nidulans* wild types as well as in the deletion mutants by qRT-PCR prior to RNA sequencing (S1 Fig), confirming the absence of transcripts in the deletion mutants.

### RNA sequencing

RNA-Seq libraries were constructed and sequenced at the Vanderbilt Technologies for Advanced Genomics Core Facility at Vanderbilt University using the Illumina Tru-seq RNA sample prep kit as previously described [28,48,50]. In brief, total RNA quality was assessed via Bioanalyzer (Agilent). Upon passing quality control, poly-A RNA was purified from total RNA and the second strand cDNA was synthesized from mRNA. cDNA ends were then blunt repaired and given an adenylated 3’ end. Next, barcoded adapters were ligated to the adenylated ends and the libraries were PCR enriched, quantified, pooled and sequenced an on Illumina HiSeq 2500 sequencer. Two biological replicates were generated for each strain sequenced.

### RNA-seq read alignment and differential gene expression

Raw RNA-seq reads were trimmed of low-quality reads and adapter sequences using Trimmomatic using the suggested parameters for single-end read trimming [87]. After read trimming, all samples contained between 20–30 million reads. The smallest sample contained 19.9 million and the largest contained 30.6 million reads; the average sample contained 24.0 million reads (S9 Table). Trimmed reads were aligned to *A*. *nidulans* and *A*. *fumigatus* genomes using Tophat2 using the reference gene annotation to guide alignment and without attempting to detect novel transcripts (parameter—no-novel-juncs) [88]. Reads aligning to each gene were counted using HTSeq-count with the intersection-strict mode [89]. Differential expression between Δ*veA* vs WT and Δ*mtfA* vs WT strains of *A*. *fumigatus* and *A*. *nidulans* were determined using the DESeq2 software, which normalizes read counts by library depth [90]. Genes were considered differentially expressed if their adjusted *P*-value was less than 0.1 and their log_2_ fold change was greater than 1 or less than-1.

### Statistical analyses

GO term enrichment was determined for over- and under-expressed genes in all four conditions tested (*A*. *nidulans* and *A*. *fumigatus* Δ*veA* vs WT and Δ*mtfA* vs WT) using the Cytoscape plugin Bingo [91,92]. To allow for a high-level view of the types of differentially expressed gene sets, the *Aspergillus* GOSlim term subset developed by *Asp*GD was used. The Benjamani-Hochberg multiple testing correction was applied, and terms were considered significantly enriched if the adjusted *P*-value was less than 0.05.

Fisher’s exact tests were performed using the R function fisher.test with a two-sided alternative hypothesis [93]. *P*-values were adjusted for multiple comparisons using the R function p.adjust with the Benjamini-Hochberg multiple testing correction [94]. Figures were created using the R plotting system ggplot2 [95] and circos [96].

## Supporting Information

S1 Fig qRT-PCR expression analysis of *veA* and *mtfA* genes in *A*. *nidulans* and *A*. *fumigatus* wild-type and deletion strains used in this study. Transcriptional pattern of *veA* and *mtfA* in the *A*. *nidulans* Δ*veA* and Δ*mtfA* strains, respectively, and corresponding control (**A,B**). *veA* and *mtfA* expression levels in the *A*. *fumigatus* Δ*veA* and Δ*mtfA* strains, respectively, and their control (**C,D**). The relative expression was calculated using 2^-ΔΔCT^ as described by Schmittgen and Livak [97]. 18S gene expression was used as internal reference. Means of three replicates are shown. Values were normalized to wild-type expression considered as 100. Error bar represents standard error.
(TIF)

S2 Fig Expression of the *dba* SM cluster genes in *A*. *nidulans* and their *A*. *fumigatus* orthologs in Δ*veA* vs WT comparisons. All heatmaps indicate the log_2_ fold change in gene expression in the Δ*veA* strain compared to the wild-type strain. Heatmap cells in blue denote genes that show lower expression in the Δ*veA* strain relative to wild-type and cells colored red denote genes with higher expression in the Δ*veA* strain. Genes listed in red font are members of SM gene clusters; genes listed in black are not.
(PDF)

S1 Table List of secondary metabolic gene clusters in *A*. *fumigatus*, *A*. *nidulans*, *A*. *niger*, and *A*. *oryzae*. (XLSX)

S2 Table Secondary metabolic gene clusters that share more than half of their orthologous genes between *A*. *fumigatus*, *A*. *nidulans*, *A*. *niger*, and *A*. *oryzae*. (XLSX)

S3 Table Genes in the *A*. *nidulans* sterigmatocystin gene cluster and their homologs in other *Aspergillus* secondary metabolic gene clusters. (XLSX)

S4 Table Conservation of secondary metabolic genes between *A*. *fumigatus*, *A*. *nidulans*, *A*. *niger*, and *A*. *oryzae* compared to conservation of genes involved in primary metabolism. (XLSX)

S5 Table Differential gene expression results for *A*. *fumigatus* and *A*. *nidulans* Δ*veA* vs WT and Δ*mtfA* vs WT comparisons. (XLSX)

S6 Table GO term enrichment analysis of genes differentially expressed in *A*. *fumigatus* and *A*. *nidulans* Δ*veA* vs WT and Δ*mtfA* vs WT comparisons. (XLSX)

S7 Table Ortholog gene counts for differentially expressed genes. Differentially expressed genes in Δ*veA* vs WT and Δ*mtfA* vs WT comparisons for *A*. *fumigatus* and *A*. *nidulans* with a focus on gene sets for secondary metabolism and development and showing the number of genes with one or more ortholog in the other species.
(XLSX)

S8 Table Primers used in this study. (XLSX)

S9 Table Number of reads passing quality control for all RNA-seq samples rounded to the nearest hundred thousand. (XLSX)

S10 Table Differential expression of genes in secondary metabolic gene clusters for *A*. *fumigatus* and *A*. *nidulans* Δ*veA* vs WT and Δ*mtfA* vs WT comparisons. (XLSX)
