# Supplementary figures and images for: Examining the Evolution of the Regulatory Circuit Controlling Secondary Metabolism and Development in the Fungal Genus Aspergillus
Source: PLoS Genet. 2015 Mar 18;11(3):e1005096. doi: 10.1371/journal.pgen.1005096 (PMC4364702; doi:10.1371/journal.pgen.1005096)

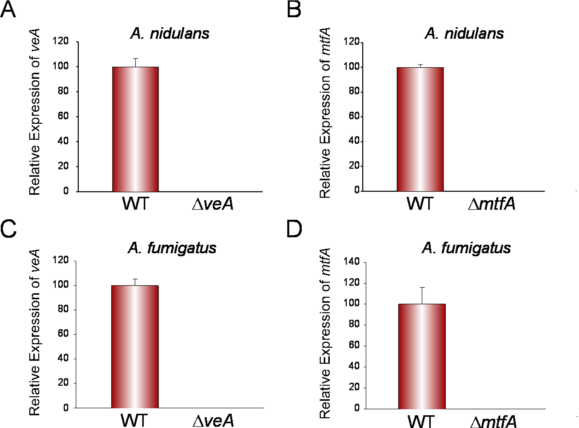

Supplement: S1 Fig — Transcriptional pattern of veA and mtfA in the A. nidulans ΔveA and ΔmtfA strains, respectively, and corresponding control (A,B). veA and mtfA expression levels in the A. fumigatus ΔveA and ΔmtfA strains, respectively, and their control (C,D). The relative expression was calculated using 2-ΔΔCT as described by Schmittgen and Livak [97]. 18S gene expression was used as internal reference. Means of three replicates are shown. Values were normalized to wild-type expression considered as 100. Error bar represents standard error. (TIF) [file pgen.1005096.s001.tif]

# Regulation of an SM cluster and its orthologs

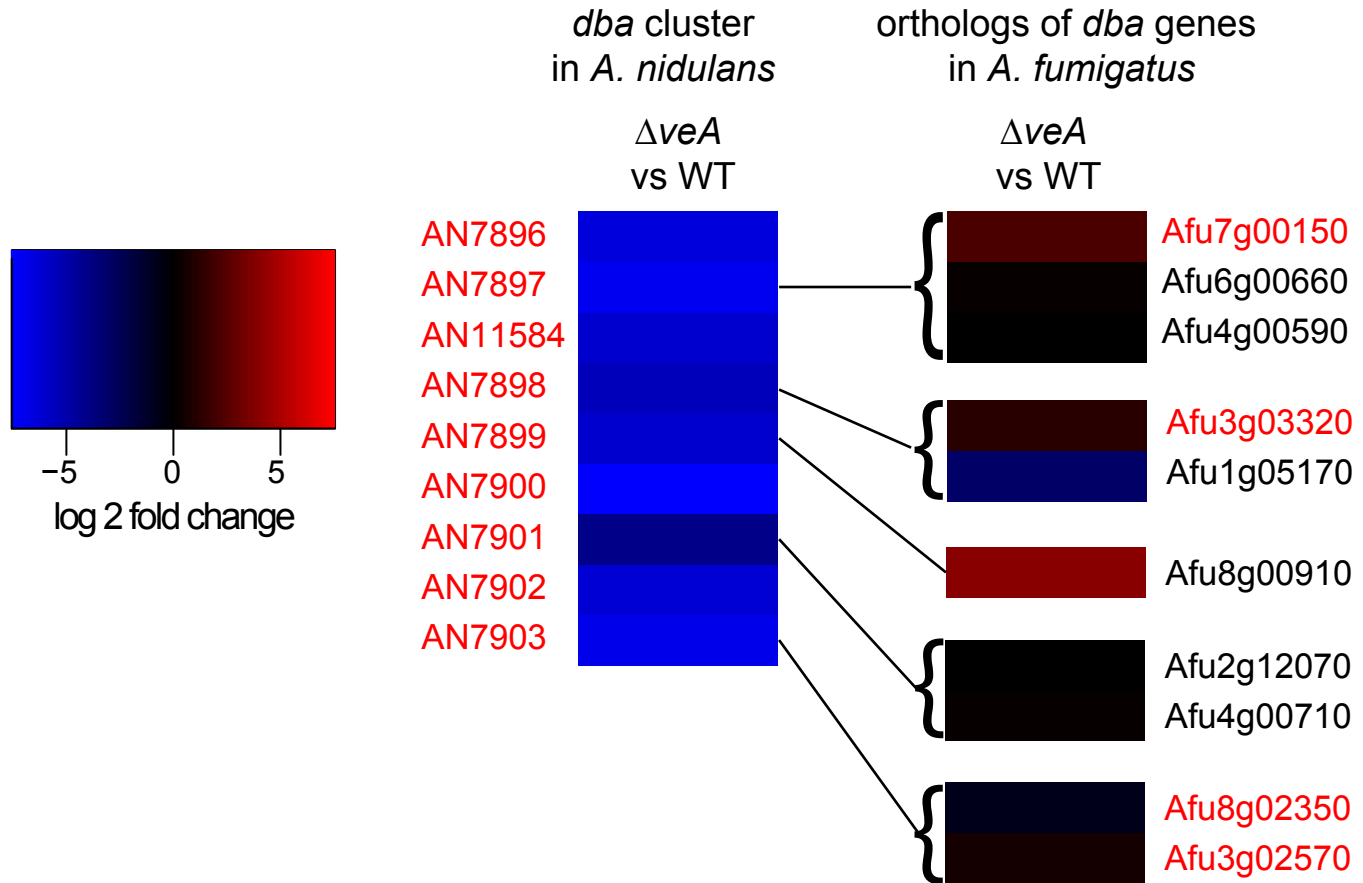

Supplement: S2 Fig — All heatmaps indicate the log2 fold change in gene expression in the ΔveA strain compared to the wild-type strain. Heatmap cells in blue denote genes that show lower expression in the ΔveA strain relative to wild-type and cells colored red denote genes with higher expression in the ΔveA strain. Genes listed in red font are members of SM gene clusters; genes listed in black are not. (PDF) [file pgen.1005096.s002.pdf]
